# Use of Tablets and Smartphones to Support Medical Decision Making in US Adults: Cross-Sectional Study

**DOI:** 10.2196/19531

**Published:** 2020-08-12

**Authors:** Aisha Langford, Kerli Orellana, Jolaade Kalinowski, Carolyn Aird, Nancy Buderer

**Affiliations:** 1 Department of Population Health, NYU Langone Health New York, NY United States; 2 Nancy Buderer Consulting, LLC Oak Harbor, OH United States

**Keywords:** smartphone, mHealth, eHealth, mobile phone, cell phone, tablets, ownership, decision making, health communication, telemedicine, monitoring, physiologic, surveys and questionnaires

## Abstract

**Background:**

Tablet and smartphone ownership have increased among US adults over the past decade. However, the degree to which people use mobile devices to help them make medical decisions remains unclear.

**Objective:**

The objective of this study is to explore factors associated with self-reported use of tablets or smartphones to support medical decision making in a nationally representative sample of US adults.

**Methods:**

Cross-sectional data from participants in the 2018 Health Information National Trends Survey (HINTS 5, Cycle 2) were evaluated. There were 3504 responses in the full HINTS 5 Cycle 2 data set; 2321 remained after eliminating respondents who did not have complete data for all the variables of interest. The primary outcome was use of a tablet or smartphone to help make a decision about how to treat an illness or condition. Sociodemographic factors including gender, race/ethnicity, and education were evaluated. Additionally, mobile health (mHealth)- and electronic health (eHealth)-related factors were evaluated including (1) the presence of health and wellness apps on a tablet or smartphone, (2) use of electronic devices other than tablets and smartphones to monitor health (eg, Fitbit, blood glucose monitor, and blood pressure monitor), and (3) whether people shared health information from an electronic monitoring device or smartphone with a health professional within the last 12 months. Descriptive and inferential statistics were conducted using SAS version 9.4. Weighted population estimates and standard errors, univariate odds ratios, and 95% CIs were calculated, comparing respondents who used tablets or smartphones to help make medical decisions (n=944) with those who did not (n=1377), separately for each factor. Factors of interest with a *P* value of <.10 were included in a subsequent multivariable logistic regression model.

**Results:**

Compared with women, men had lower odds of reporting that a tablet or smartphone helped them make a medical decision. Respondents aged 75 and older also had lower odds of using a tablet or smartphone compared with younger respondents aged 18-34. By contrast, those who had health and wellness apps on tablets or smartphones, used other electronic devices to monitor health, and shared information from devices or smartphones with health care professionals had higher odds of reporting that tablets or smartphones helped them make a medical decision, compared with those who did not.

**Conclusions:**

A limitation of this research is that information was not available regarding the specific health condition for which a tablet or smartphone helped people make a decision or the type of decision made (eg, surgery, medication changes). In US adults, mHealth and eHealth use, and also certain sociodemographic factors are associated with using tablets or smartphones to support medical decision making. Findings from this study may inform future mHealth and other digital health interventions designed to support medical decision making.

## Introduction

As of June 2019, approximately 81% of US adults owned smartphones and nearly 52% of US adults owned tablets according to the Pew Research Center [1]. By race/ethnicity, the proportion of smartphone ownership for White, Black, and Hispanic people was 82%, 80%, and 79%, respectively [1]. By gender, 79% of women and 84% of men owned a smartphone in 2019 [1]. Although smartphone and tablet ownership have increased over the past decade, the degree to which people use mobile devices to help them make medical decisions remains unclear.

A broad goal of informed decision making is to provide individuals (and family members when appropriate and desired) with the amount of understandable, accurate, and balanced information needed to make a high-quality decision about a screening, treatment, or other health-related option (eg, end-of-life issues, circumcision, vaccines, genetic tests) [2,3]. Shared decision making is a related concept to informed decision making and is broadly conceptualized as a collaborative process that allows patients and their providers to make health care decisions together, taking into account the best scientific evidence available as well as patients’ values and preferences [4]. Some studies have shown that mobile health (mHealth) and electronic health (eHealth) can improve shared decision-making opportunities and encourage greater patient participation in medical decision making [5-7]. Other studies have shown differences in who uses mHealth and eHealth by age, gender, race/ethnicity, education, and history of health conditions [8-10].

Little is known about how mHealth and eHealth tools may impact medical decision making in nonclinical, population-based samples. A better understanding of this gap in knowledge is important given that strategies to support informed decision making and shared decision making are increasingly being advocated by decision science experts and health care organizations [11-14]. The purpose of this study is to explore factors associated with self-reported use of tablets or smartphones to support medical decision making.

## Methods

### Brief Overview of the Health Information National Trends Survey

The Health Information National Trends Survey (HINTS) [15] is a probability-based, nationally representative cross-sectional survey of noninstitutionalized US adults aged 18 and over. It is the only national survey exclusively devoted to monitoring trends in health communication and the health information environment. HINTS was developed by the National Cancer Institute’s Health Communication and Informatics Research Branch and has been administered approximately every 2 years since 2003. Details about HINTS methodology are reported elsewhere [16,17] and can be seen on the HINTS website [15].

This study evaluated cross-sectional participant data from HINTS 5, Cycle 2. The sample design consisted of a single-mode postal mail survey, using the Next Birthday Method for respondent selection. Data were collected between January and May 2018. There were 3504 responses in the full HINTS 5, Cycle 2 data set; 2321 responses remained after eliminating those with missing data for any factor of interest. A total of 1183 participants were not included in this analysis: 752 respondents were removed due to unusable responses on the main outcome variable including not having a smartphone or device, 102 were removed because they replied “not applicable” to the question regarding sharing health information from an electronic monitoring device or smartphone with a health professional, and 329 were removed due to missing information on at least one of the other factors of interest (eg, demographics or medical information).

### Measures

#### Use of Tablets or Smartphones for Medical Decision Making

The primary outcome was worded as, “Has your tablet or smartphone helped you make a decision about how to treat an illness or condition?” Response options were yes/no.

#### Use of Electronic Devices Other Than Tablets and Smartphones to Monitor Health

Participants were asked, “In the past 12 months, have you used an electronic wearable device to monitor or track your health or activity? For example, a Fitbit, Apple Watch, or Garmin Vivofit.” Response options were yes/no.

#### Sharing Health Information From an Electronic Device

Participants were asked, “Have you shared health information from either an electronic monitoring device or smartphone with a health professional within the last 12 months?” Response options were yes, no, and not applicable.

#### Health and Wellness Apps

Participants were asked, “On your tablet or smartphone, do you have any ‘apps’ related to health and wellness?” Response options were yes, no, and don’t know.

### History of Medical Conditions

Participants were asked if a doctor or other health professional ever told them that they had any of the following medical conditions (yes/no): (1) diabetes or high blood sugar; (2) high blood pressure or hypertension; (3) heart condition such as heart attack, angina, or congestive heart failure; (4) chronic lung disease, asthma, emphysema, or chronic bronchitis; (5) arthritis or rheumatism; and (6) depression or anxiety disorder. Participants were also asked if they had ever been diagnosed as having cancer (yes/no)? Each medical condition was evaluated individually, as well as “at least one medical condition” compared with none.

### Demographics

Demographics such as age in years (median and categorical), gender (male and female), race/ethnicity (White, Black or African American, Hispanic, Asian, and Other), and education (<high school, high-school graduate, some college or trade school, and college graduate) were also assessed.

### Data Analysis

Weighted population estimates and standard errors were calculated to describe the sample. Odds ratios (ORs) and 95% CI were calculated, comparing respondents who used tablets or smartphones to help make medical decisions (n=944) with those who did not (n=1377), separately for each factor using univariate logistic regression models. A multivariate logistic regression model for the odds of using tablets or smartphones to make medical decisions was attempted for factors that were univariately significant with *P*<.10. CIs for ORs that do not contain 1 are considered significant for the purpose of this study. All analyses were conducted using SAS version 9.4 (SAS Institute).

## Results

Sociodemographic characteristics and univariate ORs are presented in Table 1. Briefly, categorical age, gender, mHealth, and eHealth factors were significantly associated with use of tablets or smartphones for helping participants make medical decisions. However, race/ethnicity, educational attainment, and history of various medical conditions were not significant.

In the multivariate model (Table 2), we found that men had lower odds of reporting that a tablet or smartphone helped them make a medical decision compared with women (OR 0.59, 95% CI 0.42-0.81; *P*=.002). By contrast, participants who had health and wellness apps on tablets or smartphones (OR 1.54, 95% CI 1.06-2.23; *P*=.02), used other electronic devices to monitor health (OR 1.46, 95% CI 1.08-1.97; *P*=.01), and shared information from devices or smartphones with health care professionals (OR 1.87, 95% CI 1.37-2.56, *P*<.001) had higher odds of reporting that their tablet or smartphone helped them make a medical decision, compared with those who did not. Respondents aged 75 and older had lower odds of using a tablet or smartphone compared with younger respondents aged 18-34 (OR 0.38, 95% CI 0.20-0.72).

**Table 1:** Description of population estimates and univariate odds ratios for using tablet or smartphone to help make decisions about how to treat an illness or condition.^a^

Characteristics			Use tablet/smartphone to help with medical decisions (n=944), weighted percentage (standard error)	Do not use tablet/smartphone (n=1377), weighted percentage (standard error)	Odds ratio (CI)	*P* value
**Demographics**						
	**Age (years)**					Overall .003
		18-34	28.4 (3.4)	26.8 (2.1)	Reference	
		35-49	31.2 (2.8)	30.1 (1.8)	0.98 (0.61-1.57)	.93
		50-64	31.5 (2.3)	28.8 (1.6)	1.03 (0.67-1.60)	.89
		65-74	7.0 (0.7)	9.4 (0.7)	0.70 (0.44-1.11)	.13
		75+	2.0 (0.3)	4.8 (0.5)	0.38 (0.22-0.67)	.001
	Median age (years)		45.1 (0.96)	46.2 (0.70)	0.99 (0.99-1.00)	.27
	**Gender**					
		Female	58.6 (2.4)	44.2 (1.8)	Reference	
		Male	41.4 (2.4)	55.8 (1.8)	0.56 (0.41-0.76)	<.001
	**Race ethnicity**					Overall .16
		NH^b^ White	62.7 (2.2)	68.1 (1.6)	Reference	
		NH African American	11.4 (1.2)	8.9 (0.8)	1.40 (0.98-1.99)	.06
		Hispanic	17.0 (1.7)	14.7 (1.1)	1.26 (0.87-1.80)	.21
		NH Asian	6.4 (1.5)	4.6 (0.8)	1.51 (0.65-3.48)	.33
		Other	2.5 (0.7)	3.8 (0.5)	0.73 (0.31-1.68)	.45
	**Highest level of school**					Overall .67
		College graduate+	35.0 (1.9)	31.8 (1.3)	Reference	
		Some college	41.2 (2.5)	42.6 (1.7)	0.88 (0.66-1.17)	.37
		High-school graduate	18.9 (2.3)	19.4 (1.7)	0.88 (0.57-1.36)	.57
		Less than high school	4.9 (1.1)	6.1 (1.1)	0.73 (0.38-1.41)	.35
	**Use of electronic devices other than tablets and smartphones to monitor health**					
		Yes	47.9 (2.7)	31.3 (2.0)	2.02 (1.48-2.75)	<.001
		No	52.1 (2.7)	68.7 (2.0)	Reference	
	**Share health information from an electronic monitoring device or smartphone with a health professional**					
		Yes	26.8 (1.9)	13.5 (1.4)	2.36 (1.75-3.18)	<.001
		No	73.2 (1.9)	86.5 (1.4)	Reference	
	**Presence of health and wellness apps on a tablet or smartphone**					
		Yes	61.6 (2.6)	44.3 (3.0)	2.02 (1.41-2.91)	<.001
		No or Don’t know	38.4 (2.6)	55.7 (3.0)	Reference	
**Medical conditions**						
	**Diabetes**					
		Yes	14.3 (1.2)	13.5 (1.2)	1.07 (0.81, 1.41)	.63
		No	85.7 (1.2)	86.5 (1.2)	Reference	
	**High blood pressure**					
		Yes	30.4 (1.7)	34.4 (2.4)	0.84 (0.63-1.11)	.22
		No	69.6 (1.7)	65.6 (2.4)	Reference	
	**Heart disease**					
		Yes	4.8 (0.8)	4.5 (0.8)	0.94 (0.53-1.66)	.82
		No	95.2 (0.8)	95.5 (0.8)	Reference	
	**Lung disease**					
		Yes	10.2 (1.3)	11.6 (1.3)	0.87 (0.58-1.30)	.48
		No	89.8 (1.3)	88.4 (1.3)	Reference	
	**Depression or anxiety**					
		Yes	26.3 (2.0)	22.0 (2.1)	1.26 (0.92-1.73)	.15
		No	73.7 (2.0)	78.0 (2.1)	Reference	
	**Ever had cancer**					
		Yes	7.2 (0.9)	8.2 (0.7)	0.87 (0.58-1.31)	.49
		No	92.8 (0.9)	91.8 (0.7)	Reference	
	**Arthritis**					
		Yes	19.6 (1.8)	16.9 (1.4)	1.20 (0.86-1.68)	.28
		No	80.4 (1.8)	83.1 (1.4)	Reference	
	**One or more of the above medical conditions**					
		Yes	60.2 (2.7)	60.9 (2.7)	0.97 (0.70-1.35)	.87
		No	39.8 (2.7)	39.1 (2.7)	Reference	

^a^Total number of respondents (unweighted n).

^b^Native Hawaiian.

**Table 2:** Multivariable logistic regression model for using a tablet or smartphone to help make decisions about how to treat an illness or condition.

Variable		Odds ratio (CI)		*P* value	
**Age (years)**				Overall 0.007	
	18-34		Reference		
	35-49		0.95 (0.58-1.55)		.83
	50-64		0.97 (0.60-1.56)		.90
	65-74		0.65 (0.39-1.08)		.09
	75+		0.38 (0.20-0.72)		.004
Male (vs female)		0.59 (0.42-0.81)		.002	
Use of electronic devices other than tablets and smartphones to monitor health (vs no)		1.46 (1.08-1.97)		.01	
Share health information from an electronic monitoring device or smartphone with a health professional (vs no)		1.87 (1.37-2.56)		<.001	
Presence of health and wellness apps on a tablet or smartphone (vs no or don’t know)		1.54 (1.06-2.23)		.02	

## Discussion

### Principal Findings

The goal of this study was to explore factors associated with self-reported use of tablets or smartphones to support medical decision making in a large, nationally representative sample of US adults. In summary, we found that some sociodemographic factors including categorial age and gender, presence of health and wellness apps, and use of mHealth or eHealth tools to monitor health were associated with using a tablet or smartphone to support medical decision making. Although sociodemographic differences in tablet and smartphone use are well documented [8,18,19], this study is the first-known investigation on the use of tablets or smartphones in the specific context of medical decision making.

Over the last decade, there has been growing use of mHealth and eHealth interventions. Notably, the more contemporary term *digital health* includes categories such as mHealth, health information technology, wearable devices, telehealth and telemedicine, and personalized medicine [20]. Our findings complement prior studies exploring various aspects of mHealth and eHealth to support patients’ behavior change and self-management across health conditions—often done via telemedicine, SMS text messaging, or smartphone apps [21-26]—as well as studies showing that tablets and smartphones can help support chronic disease management [18]. Novel uses of mobile devices are being explored to support shared decision via *digital phenotyping* [27] or “moment-by-moment quantification of the individual-level human phenotype in-situ using data from smartphones and other personal digital devices” [28]. While it has been established that people with more than one health condition are more likely to use digital health tools [29,30], we did not observe an association between the number and type of health conditions with the use of tablets or smartphones to support medical decision making. Moreover, we did not observe an effect of race/ethnicity or educational attainment with regard to using tablets or smartphones to support medical decision making.

A key finding from this study was that HINTS participants aged 75 and older had lower odds of using tablets or smartphones compared with participants aged 18-34. While this finding is not surprising given that older US adults generally have lower rates of tablets and smartphones ownership compared with their younger counterparts, it should not be interpreted as unwillingness or inability of people aged 75 or older to use mobile devices. For example, Parker et al. [31] found that 85% of adults in their study aged 60 years or older were willing to try mHealth devices. In that same study, barriers to mHealth use included concerns about cost and unfamiliarity with technology, while facilitators included prior training on how to use mHealth devices and devices tailored to the functional needs of older adults [31]. In a different study, Seifert et al. [32] explored the willingness of adults aged 50 years or older to share mobile health data with researchers and found that approximately 57% were willing to do so.

Moving forward, increased use of mobile devices to support decision making may be realized as more trainings on how to use these tools and resources to lessen the financial burden of getting these devices become available in community and health system settings [33]. Additionally, as future research studies about digital health include more older adults, researchers will have a better understanding of the information needs and digital design preferences of this group. For example, user-centered design processes can help inform how design choices made in the development stages may positively or negatively affect medical decision making in older adults.

With regard to gender, we found that men had lower odds of using tablets or smartphones to support medical decision making compared with women. While somewhat speculative, it is possible that women use tablets and smartphones more often than men to support medical decision making for at least three reasons. First, women may be more likely than men to manage health-related decisions for family members including children, parents, and partners. Second, women may be more likely than men to make and keep routine health care appointments, which in turn, may present more opportunities for medical decision making. Third, women may have more health-related decisions to make due to physiology. For example, women often make choices related to birth control, childbirth, breastfeeding, breast and cervical cancer screening and related treatment, breast reconstruction after a mastectomy, menopause, and uterine fibroids. Not surprisingly, we also found that the use of other electronic devices beyond tablets and smartphones to monitor health (eg, Fitbit, blood glucose meters, and blood pressure monitors), the presence of health and wellness apps on a tablet or smartphone, and sharing information from an electronic monitoring device or smartphone with a health professional were associated with the use of tablets or smartphones to support medical decision making. These findings may be partially explained by the notion that people who are comfortable using mobile devices may have higher levels of eHealth and digital health literacy compared with those who do not use these tools, and are therefore more likely to use mHealth tools to support decision making.

**Figure 1:**
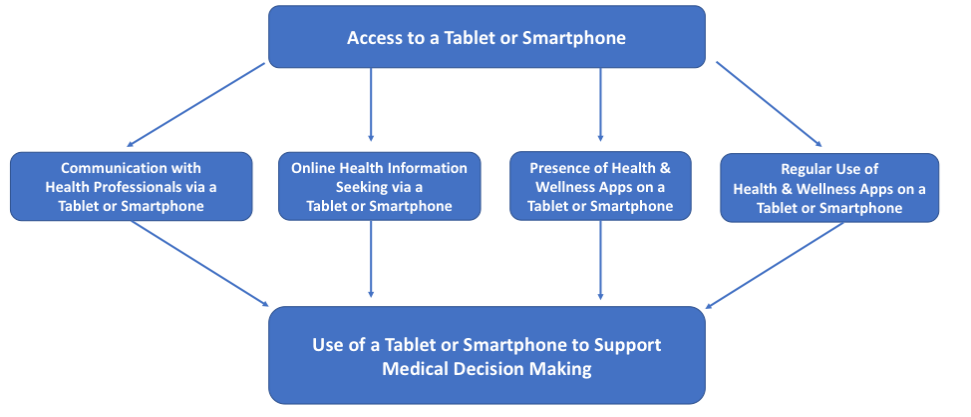
Conceptualization of How Mobile Devices May Help Support Medical Decision Makingd.

There are several plausible pathways by which tablets or smartphones can help support medical decision making. As shown in Figure 1, we offer 4 potential explanations. It should be noted that our starting point is access to a tablet or smartphone. We specifically use the term *access* to a tablet or smartphone instead of *ownership* because some clinical research projects provide participants with mobile devices as part of a study. Additionally, some people may use another person’s mobile device, particularly if they are in the same household.

First, it is possible that people use their tablets or smartphones to communicate with health professionals. For example, people may call, email, send SMS text messages, and use apps or other platforms to have virtual face-to-face visits with health professionals. People may also share health information from an electronic monitoring device or smartphone with health professionals. These communication opportunities may help patients get their health-related questions and concerns addressed, which in turn can help them make a medical decision. Second, people may use tablets or smartphones for online health information-seeking purposes [34-38]. In 2016, approximately 36.6 million online users in the US reported that they accessed the internet exclusively via mobile devices according to Statista [39]. Furthermore, in 2019, approximately 74% of US adults reported that they accessed the internet daily via a mobile device according to HINTS data [40]. It is possible that people are using their tablets or smartphones to search for health information online to help them make a medical decision. On the one hand, these online searches may take people to reputable sources of health information such as the Centers for Disease Control and Prevention (CDC), National Institutes of Health (NIH), MedlinePlus [41], disease-specific organizations (eg, American Heart Association), or the Ottawa Patient Decision Aid Inventory [42]. On the other hand, online health information seeking may also expose people to misinformation and biased sources of health information [36]. Nevertheless, regardless of the actual or perceived quality of health information found online, it may impact how individuals make medical decisions for themselves or a loved one. Third, it is possible that the presence of health and wellness apps on a tablet or smartphone may impact how a person makes a decision about how to treat a health condition or illness, even if the app is used infrequently or one time. For example, some patient portals are now available as mobile apps (eg, MyChart in Epic). Through these apps, patients can access their medical records online and see laboratory results, clinical notes, and medication lists. It is possible that seeing a one-time laboratory result is enough to initiate medical decision making. For example, if a person gets a laboratory report showing high triglycerides, they may decide to make lifestyle changes or consider taking prescription medications. Fourth, regular use of health and wellness apps may also support medical decision making. For example, some patients use health and wellness apps regularly to (1) document home readings for conditions such as stage 1 hypertension and type 2 diabetes; (2) track symptoms such as pain, cognitive problems, or disrupted sleep; and (3) monitor health behaviors such as physical activity and diet. The data gathered from regular use of health and wellness apps may inform future decisions about how to treat a health condition or an illness.

**Strengths, Limitations, and Future Directions**

Strengths of this study include the use of HINTS, a nationally representative survey designed to track health communication and health information technology, and the study’s focus on the role of tablets and smartphones for medical decision making. Despite these strengths, our results should be interpreted in the context of both known and potential limitations. For example, we do not know the specific medical decisions that participants were referring to when completing the survey (eg, to pursue surgery, start or change medications, get genetic testing). Based on the wording of the HINTS question about health and wellness apps on a tablet or smartphone, we are not able to distinguish between the simple *presence* of apps and whether or not participants *used* these apps on a regular basis. Finally, while we offered some explanations in Figure 1 regarding plausible ways by which mobile devices may support decision making, we do not know all of the potential mechanisms by which tablets or smartphones may help support medical decision making.

Future research should explore how, if at all, frameworks such as the Ottawa Decision Support Framework [43] and models such as Technology Acceptance Model may help explain our findings [44]. Related, more research is needed to understand the various mechanisms by which electronic wearable devices to monitor health (ie, activity trackers or blood pressure monitors) and the sharing of information from these devices with health professionals may have an impact on subsequent medical decision making and behavioral outcomes such as cancer screening, weight management, and dietary patterns. Future work should also evaluate which attributes of health and wellness apps have the biggest impact on reported use of tablets and smartphones to support medical decision making. For example, the use of icon arrays and pictographs to convey risk, sixth grade or less level readability of text, use of values clarification methods, and a balanced presentation of health care options are commonly used principles in print and web-based decision aids [45,46]. However, the literature on how to incorporate these principles into health and wellness apps is less developed. Finally, future studies should evaluate the role of digital health literacy and general health literacy in the context of using mobile devices to support medical decision making [47].

This manuscript is especially timely given that it was written in April 2020 as the coronavirus pandemic was spreading throughout the US. Due to social distancing recommendations and widespread cancelations of nonurgent, in-person medical appointments [48,49], there is now even greater attention given to the importance of mHealth and eHealth with regard to (1) conducting telehealth visits and ensuring equitable access to racial/ethnic and other sociodemographic groups, (2) supporting informed decision making about participation in clinical trial for COVID-19 (coronavirus 2019), and (3) shared decision making between patients and clinicians about how to manage chronic conditions for patients who are concerned about being seen in-person as clinics begin to open.

### Conclusion

Tablets and smartphones hold promise for supporting medical decision making and may provide new opportunities for facilitating prevention, early diagnosis of diseases, and management of chronic conditions outside of traditional health care settings [20]. Moreover, mobile technologies are prevalent in minority communities and may serve as an important bridge to inclusivity and access to resources, thereby enhancing opportunities for informed and shared decision making [50,51]. Given the growing use of digital health tools available to the general public (eg, health and wellness apps), digital health technology in clinical practice, and focus on supporting informed and shared decision for US adults, data on the role of tablets and smartphones in supporting medical decision making are important to track over time.

## Abbreviations

CDC: Centers for Disease Control and Prevention
HINTS: Health Information National Trends Survey
NIH: National Institutes of Health
OR: odds ratio
